# The Influence of Ethanolic Extract of Brazilian Green Propolis Gel on Hygiene and Oral Microbiota in Patients after Mandible Fractures

**DOI:** 10.1155/2016/9190814

**Published:** 2016-08-10

**Authors:** Iwona Niedzielska, Zbigniew Puszczewicz, Anna Mertas, Damian Niedzielski, Bartosz Różanowski, Stefan Baron, Tomasz Konopka, Agnieszka Machorowska-Pieniążek, Małgorzata Skucha-Nowak, Marta Tanasiewicz, Jarosław Paluch, Jarosław Markowski, Bogusława Orzechowska-Wylęgała, Wojciech Król, Tadeusz Morawiec

**Affiliations:** ^1^Department and Hospital of Craniomaxillofacial Surgery and Oral Surgery, School of Medicine with the Division of Dentistry in Zabrze, Medical University of Silesia in Katowice, Plac Akademicki 17, 41-902 Bytom, Poland; ^2^Department of Microbiology and Immunology, School of Medicine with the Division of Dentistry in Zabrze, Medical University of Silesia in Katowice, Jordana 19, 41-808 Zabrze, Poland; ^3^Institute of Oral Medicine, Medical University of Lodz, Pomorska 251, 92-957 Łódź, Poland; ^4^Pedagogical University of Cracow, Podchorążych 2, 30-084 Kraków, Poland; ^5^Chair of Masticatory Dysfunction and Orthodontics, School of Medicine with the Division of Dentistry in Zabrze, Medical University of Silesia in Katowice, Plac Traugutta 2, 41-800 Zabrze, Poland; ^6^Department of Periodontology, Wrocław Medical University, Krakowska 26, 50-425 Wrocław, Poland; ^7^Department of Orthodontics, Chair of Masticatory Dysfunction and Orthodontics, School of Medicine with the Division of Dentistry in Zabrze, Medical University of Silesia in Katowice, Plac Traugutta 2, 41-800 Zabrze, Poland; ^8^Department of Conservative Dentistry with Endodontics, School of Medicine with the Division of Dentistry in Zabrze, Medical University of Silesia in Katowice, Plac Akademicki 17, 41-902 Bytom, Poland; ^9^Hospital of Laryngology, Medical University of Silesia in Katowice, Francuska 20/24, 40-055 Katowice, Poland; ^10^Department of Oral Surgery, School of Medicine with the Division of Dentistry in Zabrze, Medical University of Silesia in Katowice, Plac Akademicki 17, 41-902 Bytom, Poland

## Abstract

Maintenance of proper oral hygiene by dental plaque elimination is one of the most important factors affecting the healing process in postoperative oral wounds. Propolis is a substance produced by bees. Ethanolic extract of propolis has bactericidal, fungicidal, anti-inflammatory, and antioxidative properties. Moreover, it can scavenge free radicals. The purpose of this paper is to demonstrate the efficacy of a gel containing 3% of ethanolic extract of Brazilian green propolis (EEP-B) when used for maintaining oral hygiene in patients with postoperative oral mucosal wounds. The hygiene was assessed using API, OHI, and SBI followed by microbiological examinations. The patients were divided into two groups. Group 1 consisted of those who used a gel containing EEP-B for oral hygiene, and group 2 consisted of those who used a gel without EEP-B. Although improved oral hygiene was noted in both groups, the improvement was markedly greater in the group using gel containing EEP-B. Summing up the results of microbiological examinations, EEP-B has beneficial effect on mouth microflora in postoperative period. Propolis preparations used for oral hygiene allow eliminating microorganisms of pathogenic character and physiological flora microorganisms considered as being opportunistic, with no harmful influence on physiological microflora in oral ecosystem.

## 1. Introduction

The number of craniofacial fractures has been increasing dramatically due to progress in civilization and development of motor transport accompanied by haste, stresses, and aggression. Although the mandible or inferior maxillary bone is highly resistant to bending or stretching, it is particularly prone to central or lateral injuries due to its anatomical position and lack of protection by other bones. Mandible fractures constitute 51–69% of all craniofacial fractures [1–6], affecting men 4-5 times more often than women [2, 5, 6] and occurring usually between third and fourth decades of life [5]. A majority of mandible fractures occur as a result of assault (48–65%), whereas traffic accidents and falls take further places [4–6]. The mandible frequently breaks in the body region, rarely in the condloid process, and very rarely in the median body, ramus, or coronoid process [2, 5]. Mandible fractures can be complete or incomplete, open or closed, and single, double, or comminuted and can be made through direct or indirect mechanism. There are three commonly used methods of treating mandible fractures (conservative/orthopedic, surgical/orthopedic, and surgical).

In recent years, a method of choice has been miniplate osteosynthesis. If properly stable, the method eliminates the necessity to use intermaxillary immobilization based on standard splints, thus preserving the function of temporomandibular joints and stomatognathic system muscles and enabling better eating conditions or oral hygiene [7–10]. However, oral hygiene is often found bad or insufficient irrespective of the treatment method applied. Pharmaceutical market offers a variety of drugs and preparations (mouthwashes, ointments, etc.) to help the patients maintain proper oral hygiene. It seems obvious that correct tooth brushing and the use of correct toothpaste are essential. Therefore, an attempt to seek an optimal toothpaste for a selected group of patients was made.

Propolis is a wax/resin mixture used by bees to seal up holes and slits in the beehive. Its origin is not quite clear. Probably it is collected by bees from tree buds or other botanical sources or it may be a pollen product secreted by bees as indigestible. Propolis is a hard resin mass, softening when heated to over 40 degrees centigrade and melting at 80–104 degrees centigrade. It dissolves in ethanol or ether and does not dissolve in water. The composition is complex and varies according to its origin [11–13]. Propolis usually contains resins (40%), waxes (23–30%), polyphenols (14–16%), polysaccharides (2.5%), volatile matters (>10%), and mechanical impurities. A number of preparations, showing biological activity, have been obtained through organic solvent extraction. Among solvents, ethanol is most commonly used, and ethanolic extract of propolis (EEP) has wide practical applications [14, 15]. Propolis extracts have successfully been used so far in pyodermitis caused by bacteria or fungi, furunculosis, eczemas, trophic tibial ulcers, decubitus ulcers, and endocervicitis. Therapeutic effects of EEP have also been noted in orthopedics when bone fillings containing EEP are used for treatment of bone marrow inflammatory conditions. Moreover, good results are obtained in oral medicine, in cases of dry sockets and parodontopaties [16, 17]. In clinical applications, EEP is shown to have regenerative effects, and this observation is confirmed by a number of experiments. EEP is widely reported as having bactericidal, fungicidal, anti-inflammatory [12, 18–24], and anticarcinogenic [25–28] properties. Other authors report that EEP can scavenge free radicals and act antiparasitically or as an antioxidant [23]. Also, EEP can act against viruses like* Toxoplasma gondii* an* Trichomonas vaginalis* [14]. EEP has been shown to have inhibitory effect on* Candida albicans* [29] and to have synergistic activity with some antibiotics [30, 31]. It seems that beneficial effects of propolis and honey are probably connected with the presence of flavonoids, stilbenes, and phenolic acids and their esters [12, 30, 32]. Brazilian propolis was classified into 12 groups based on their physicochemical characteristics [33]. The propolis type most commercialized is known as “green propolis” and it has been extensively studied and used in food and beverages. The botanical origin of propolis group 12 was the resin of* Baccharis dracunculifolia* in southeastern Brazil [34, 35].

Nowadays, the propolis extract is used as an addition to oral care preparations (toothpastes, mouthwashes, and prophylactic gels) enhancing their antibacterial, disinfecting, and anti-inflammatory effects [36–38]. No reports have been found in the literature on the relationship between oral hygiene and the healing process in patients after mandible fractures. However, much consideration is given to oral hygiene in other oral conditions, including parodontopaties [39, 40]. The purpose of our study is an attempt to assess prophylactic efficacy of Brazilian green propolis tooth gel used for oral hygiene after mandible fractures.

## 2. Material and Methods

### 2.1. Propolis

Propolis samples were obtained from colonies of Africanized honeybees (*Apis mellifera*), from the beekeeping section of the Seiri Alimentos Naturais Brazil in Minas Gerais State, southeastern Brazil. The twelve distinct groups of Brazilian propolis have been classified according to their botanical origin and biological properties: five from the south, six from the northeast, and one from the southeast named propolis “green” [33, 41]. The studied green propolis from Minas Gerais State in southeast Brazil was derived mainly from alecrim plants* Baccharis dracunculifolia *(Asteraceae), which are present in the field vegetation in Brazil as large populations of* Baccharis* species [34]. The unprocessed Brazilian green propolis was sent to the Nihon Natural Therapy Co. Ltd. (Tokyo, Japan) for preparation of the EEP. Propolis was extracted for 4 days in 95% V/V ethyl alcohol at 37°C, in a hermetically sealed glass vessel under occasional shaking. The ethanolic extract of Brazilian green propolis (EEP-B) was then filtered and evaporated under reduced pressure at 60°C. Previously described chemical evidence of EEP-B, based on high-performance liquid chromatography (HPLC-DAD) analysis, suggested that the main flavonoid compounds presented in studied EEP-B were kaempferol and quercetin, as well as other ingredients, such as cinnamic acid derivatives:* p*-coumaric acid and artepillin C [37, 42]. The tooth gel with 3% EEP-B (CA gel) and without EEP-B (CC gel,* placebo*), used in this study, was prepared in Nippon Zettoc Co., Ltd. (Tokyo, Japan), especially for our research. The CC gel was the gel base containing no active ingredients, which after 3% EEP-B addition became CA gel used in our study.

### 2.2. Patients

The study included 31 patients (24 men and 7 women) treated in Hospital of Maxillofacial Surgery, Medical University of Silesia in Katowice, for mandible fractures by stable osteosynthesis. The criteria for inclusion were mature age, condition after stable osteosynthesis where the fixation was performed intraorally, and teeth preserved in all sextants of the mouth. All patients were informed on the purpose of the study and agreed to participate in it. The research program was approved by the Bioethics Committee of the Silesian Chamber of Medicine (resolution number 6/2010, dated 01.03.2010).

In our study, the distribution of patients to the study group was made at random. The patients were divided into two study groups: study group CA that consisted of 16 patients (14 men and 2 women) who used a tooth gel containing 3% EEP-B for oral hygiene (CA gel) and study group CC that consisted of 15 patients (10 men and 5 women) who used a tooth gel without EEP-B for oral hygiene (CC gel,* placebo*). The results were examined on days 1, 8, and 22 following the surgery. For the purpose of the study, both clinical and microbiological examinations were performed.

### 2.3. Clinical Protocol

Clinical examinations consisted of case history, assessment of the mouth including dentition state, and assessment of oral hygiene condition. On days 1 and 22, a sample for microbiological testing was taken from the mouth floor mucosa using sterile swab. Case history was made by completing a short written questionnaire about oral hygiene habits, frequency of visits to the dentist's, or social and economic status. Oral hygiene was assessed based on Approximate Plaque Index (API) [43] and Oral Hygiene Index (OHI) [44]. Parodontium was assessed using Sulcus Bleeding Index (SBI) [45]. At the first visit (day 1 after the surgery), the history was taken and clinical examination was performed, the latter including assessment of dentition and assessment of oral hygiene using API together with simplified OHI for dental plaque and dental calculus. Also, assessment of parodontium (using SBI) and of mucosa was performed. A sample was taken from the mouth floor mucosa for microbiological testing. The patient received hygienic aids and was instructed as to oral hygiene and how to brush the teeth by Fones' method, in which positioning the brush is perpendicular to the long axis of the tooth. After suitable positioning is performed, circular rotating movements with compact dental arches were performed, also when cleaning the surface of the cheek and lip and other areas of the oral cavity. At the second visit (day 8 after the surgery), the history was taken with special attention to oral hygiene. Clinical examination included assessment of oral hygiene using API and simplified OHI for dental plaque and dental calculus, assessment of parodontium using SBI, and assessment of mucosa. At the third visit (day 22 after the surgery), the history was taken with special attention to how many times per day the teeth were treated according to instructions. Clinical examination included assessment of dentition using PUW index, assessment of oral hygiene using API together with simplified OHI for dental plaque and dental calculus, assessment of parodontium using SBI, and assessment of mucosa. A sample was taken from the mouth floor for microbiological testing. The patients were asked to complete a questionnaire about the quality of the gel used throughout the study.

### 2.4. Microbiological Investigation

Microbiological investigations were performed in the Department of Microbiology and Immunology in Zabrze, Medical University of Silesia in Katowice. The material for microbiological testing was inoculated on suitable culture media (Columbia agar, Schaedler K3 agar, and Sabouraud agar) from Biomerieux (Marcy-l'Etoile, France). Aerobic bacteria were propagated on Columbia agar medium with 5% sheep blood at 37°C. Anaerobic bacteria were propagated on Schaedler K3 medium with 5% sheep blood at 37°C in anaerobic conditions using Genbag anaer (Biomerieux, Marcy-l'Etoile, France).* Candida* fungi were propagated on selective Sabouraud agar medium at 35°C in aerobic conditions. Upon isolation and further culture of each microorganism, their species were identified with the help of the following reagent sets: ENTEROtest 24N, NEFERMtest 24N, STREPTOtest 24, STAPHYtest 24, ANAEROtest 23 (Erba-Lachema, Brno, Czech Republic), and Api Candida (Biomerieux, Marcy-l'Etoile, France).

### 2.5. The Statistical Analysis

The STATISTICA version 10 software (StatSoft, Cracow, Poland) was used to perform the statistical analysis. The statistical differences between CA and CC subgroups were determined by analysis of variance followed by Student's *t*-test (the results correlated with a normal distribution). Differences between the mean values were considered to be statistically significant at *p* ≤ 0.05.

## 3. Results

Final study group consisted of 31 patients treated for mandible fractures by stable osteosynthesis, in which study group CA (CA gel with 3% EEP-B) included 16 patients and study group CC (CC gel without EEP-B) included 15 patients. The highest mean level of API was noted at the first visit (API I) in patients using CA gel and the lowest was noted at the third visit (API III) in the same group of patients. The highest mean level of OHI was noted at the first visit (OHI I) in patients using CA gel and the lowest was noted at the third visit (OHI III) in patients using CC gel. The highest mean level of SBI was noted at the first visit (SBI I) in patients using CA gel and the lowest was noted at the third visit (SBI III) in patients using CA gel (Table 1). The first assessment of oral hygiene condition (based on API, OHI, and SBI) was performed in the first day after surgery, when the studied patients could not abide by hygiene regime of oral cavity due to the significant pain after surgery. This may be the cause of the observed highest values of API, SBI, and OHI at the first examination of studied patients.

**Figure 1:**
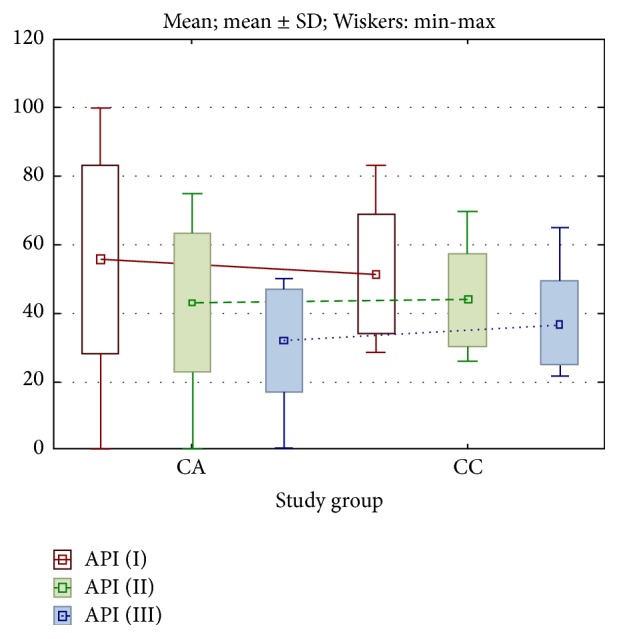
Changes in API parameter in studied groups of patients. I: first visit, day 1 after the surgery (before CA or CC gel application, baseline). II: second visit, day 8 after the surgery. III: third visit, day 22 after the surgery (following CC or CA gel application, final assessment).

In study group CA, the mean value of API parameter was statistically significantly highest at the first visit and the differences were statistically significant between first and second, between first and third, and between second and third visits (*p* < 0.0001) (Figure 1).

In study group CC, the mean value of API parameter was also statistically significantly highest at the first visit, and the differences were statistically significant between first and second, between first and third, and between second and third visits (*p* < 0.0001) (Figure 1).

**Figure 2:**
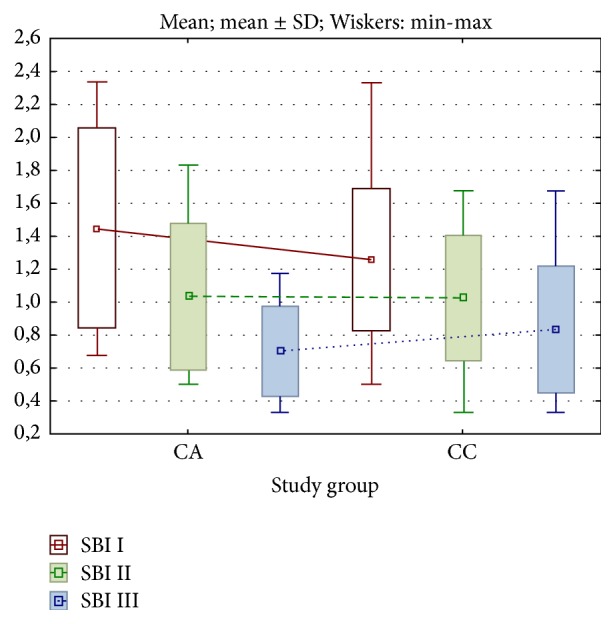
Changes in SBI parameter in studied groups of patients. I: first visit, day 1 after the surgery (before CA or CC gel application, baseline). II: second visit, day 8 after the surgery. III: third visit, day 22 after the surgery (following CC or CA gel application, final assessment).

In study group CA, the mean value of SBI parameter was statistically significantly highest at the first visit, and the differences were statistically significant between first and second, between first and third, and between second and third visits (*p* < 0.001) (Figure 2).

In study group CC, the mean value of SBI parameter was also statistically significantly highest at the first visit, and the differences were statistically significant between first and second, between first and third, and between second and third visits (*p* < 0.001) (Figure 2).

**Figure 3:**
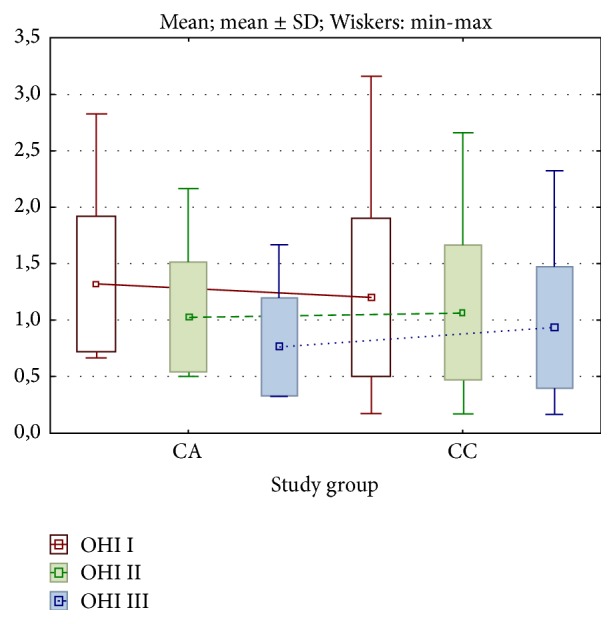
Changes in OHI parameter in studied groups of patients. I: first visit, day 1 after the surgery (before CA or CC gel application, baseline). II: second visit, day 8 after the surgery. III: third visit, day 22 after the surgery (following CC or CA gel application, final assessment).

In study group CA, the mean value of OHI parameter was statistically significantly highest at the first visit, and the differences were statistically significant between first and second, between first and third, and between second and third visits (*p* < 0.001) (Figure 3).

In study group CC, the mean value of OHI parameter was also statistically significantly highest at the first visit, and the differences were statistically significant between first and second, between first and third, and between second and third visits (*p* < 0.005) (Figure 3).

The results of microbiological examinations revealed significant differences, both qualitative and quantitative, in oral microbiota composition among patients who used CA gel with 3% EEP-B for everyday oral care (Tables 2 and 3), as compared with patients using CC gel without EEP-B (Tables 4 and 5). The differences in the number of the microorganisms species and strains, isolated from mouth floor mucosa of patients who used gel with or without EEP-B (specimen CA or CC), were presented in Table 6.

## 4. Discussion

Mandible fractures are most commonly treated by miniplate osteosynthesis, which enables better oral hygiene after mandible fractures compared with traditional conservative or orthopedic methods. Inadequate oral hygiene may lead to fracture crevice infection and finally result in osteitis [46]. During preliminary stages of the healing process postoperatively, the patients urgently need careful oral hygiene due to possible postoperative complications like lockjaw, edema, or pains.

A serious problem with this group of patients is insufficient or inadequate oral hygiene. This is often made up by the hospital staff who quickly demonstrate or instruct how to care for oral hygiene and the wound. So far no attention has been given, due to financial restrictions in this group, to what kind of oral cleaners should be used. Therefore, a suggestion as to using various oral preparations, including those containing propolis, seemed important [38, 47, 48]. Antibacterial activity of propolis is complex and not quite clear [49]. Mirzoeva et al. [50] showed that the effects of propolis activity were dependent on species and closely related to degradation of the bacterial cell membrane due to its higher permeability by ions. As a consequence, such cell may lose its membrane potential losing the motility and virulence. Other investigators suggest that antibacterial properties of propolis may be connected with some additional mechanisms like inhibition of glucosyltransferase synthesis or production of Streptococci polysaccharides [51, 52].

Skaba et al. described in their studies a beneficial effect of propolis gel in patients with healthy parodontium and those with parodontitis [37]. In our study, the final examination after 24 days of CA or CC gel use for oral hygiene showed that the patients who used CA gel with 3% EEP-B had better oral hygiene and parodontal health state. The changes were statistically significant in both groups (for API *p* < 0.0001, and for SBI *p* < 0.001). In case of OHI index, the changes were also statistically significant in case of CA gel user, *p* < 0.001, and for CC gel user, *p* < 0.005.

As revealed by the results of our examinations, the composition of oral microbiota changed considerably after CA gel with 3% EEP-B had been used, compared to patients using CC gel without EEP-B (Tables 2 [Table tab3] [Table tab4] [Table tab5]–6). The analysis of a relationship between tooth gel with EEP-B (CA preparation) and mouth microbiota after mandible fractures revealed beneficial qualitative changes in its composition consisting of elimination of potential bacterial pathogens and maintaining normal physiological flora composition.

Among ten patients using gel with 3% EEP-B (CA preparation), a similar number of microorganism isolates were detected by both first and second microbiological examinations of the swabs from mouth floor mucosa. The first examination (day 1 after surgery) revealed 54 microorganism isolates representing 28 species, and next examination (day 24 after surgery) revealed 48 microorganism isolates representing 20 species (Table 6). The final examination (after three weeks of using CA gel) revealed that 5 species of microorganisms had been eliminated (*Clostridium perfringens*,* Actinomyces naeslundii*,* Prevotella bivia*,* Bifidobacterium breve*, and* Staphylococcus epidermidis MSCNS*), whereas 7 new species appeared in the mouth microbiota (*Streptococcus vestibularis*,* Veillonella parvula*,* Bifidobacterium adolescentis*,* Bifidobacterium dentium*,* Bifidobacterium longum*,* Lactobacillus acidophilus*, and* Fusobacterium nucleatum*). Also, final examination showed a smaller amount of* Streptococcus mitis*,* Streptococcus salivarius*, and* Escherichia coli* isolates as compared with the first examination and an increased amount of* Neisseria* sp. isolates. In case of the other isolated microorganisms of physiological microbiota (*Streptococcus sanguis*,* Propionibacterium propionicus*) and microorganisms of temporary microbiota (*Streptococcus pneumoniae*,* Sarcina *sp.), an identical number of the isolates was detected by both microbiology examinations.

On the other hand, no such beneficial changes were observed in the group of patients who used CC gel without EEP-B (CC preparation) for oral hygiene. After three weeks of using CC gel without EEP-B, an increased number of microorganism isolates were detected. The first examination (day 1 after surgery) revealed 41 isolates of 18 species, and the next examination (day 24 after surgery) revealed 57 isolates representing 25 different species. Also, final examination revealed elimination of 3 microorganism species (*Bifidobacterium dentium*,* Propionibacterium acnes*, and* Escherichia coli*) and enrichment of the mouth microbiota by 16 new species, chiefly Gram(+) micrococcus types (*Streptococcus oralis*,* Streptococcus salivarius*,* Streptococcus vestibularis*,* Staphylococcus aureus MSSA*,* Peptostreptococcus prevotii*,* Ruminococcus productus*, and* Sarcina *sp.) and Gram(−) micrococcus types (*Veillonella parvula*), as well as Gram(+) rods (*Bifidobacterium adolescentis*,* Lactobacillus acidophilus*), Gram(−) rods (*Bacteroides ureolyticus*,* Fusobacterium mortiferum*,* Fusobacterium nucleatum*, and* Mitsuokella multiacidus*), and Gram(+) filamentous bacteria (*Actinomyces israelii*,* Actinomyces odontolyticus*). And finally examination of patients using CC gel without EEP-B revealed a smaller number of* Streptococcus mitis* and* Candida albicans* isolates, and a larger number of* Streptococcus sanguinis* and* Neisseria *sp. isolates. Similar changes in oral microbiota composition were noted by Morawiec et al. [38]. Tanasiewicz et al. [36] have demonstrated that preparations (toothpastes, gels) containing 3% ethanol extract of propolis seem to encourage dental plaque reduction and have a therapeutic effect on marginal parodontium. These findings are similar to ours as we observed a qualitative and quantitative change in bacterial microflora. Summing up the results of the microbiological examinations, EEP-B has a beneficial effect on the mouth microbiota in postoperative period due to its specific properties. The use of propolis in oral hygiene preparations allows eliminating pathogenic microorganisms and other microorganisms considered as being opportunistic pathogens from the mouth physiological microbiota and preserving the oral microbiota in safe condition.

Propolis offers many benefits, but its use may bring the risk of an allergy [17]. In light of the increasing antibiotic-resistance and drug-resistance, as observed nowadays, it seems reasonable to seek an alternative to bactericidal or bacteriostatic drugs among the methods offered by alternative or complementary medicine. Propolis preparations used for oral hygiene seem, therefore, to be a beneficial alternative to preparations containing chlorhexidine or triclosan.

## Figures and Tables

**Table 1:** Mean values and standard deviations (SD) of oral hygiene (OHI, API) and sulcus bleeding indexes (SBI) in studied groups of patients.

Parameter	Study group CA (*n* = 16)			Study group CC (*n* = 15)		
	Average	(Range)	SD	Average	(Range)	SD
API I	55.69	(42–100)	27.50	51.31	(28.5–83)	17.44

API II	43.02	(31–75)	20.35	43.95	(26–69.5)	13.56

API III	31.85	(23–50)	14.89	36.98	(21.7–65)	12.36

SBI I	1.32	0–0.5 (0%)	0.60	1.21	0–0.5 (0%)	0.70
		0.5–1.5 (56%)			0.5–1.5 (73%)	
		1.5–2.5 (44%)			1.5–2.5 (27%)	

SBI II	1.03	0–0.5 (0%)	0.49	1.21	0–0.5 (7%)	0.59
		0.5–1.5 (75%)			0.5–1.5 (73%)	
		1.5–2.5 (25%)			1.5–2.5 (20%)	

SBI III	0.77	0–0.5 (19%)	0.44	0.94	0–0.5 (13%)	0.54
		0.5–1.5 (81%)			0.5–1.5 (80%)	
		1.5–2.5 (0%)			1.5–2.5 (7%)	

OHI I	1.45	0–0.5 (0%)	0.61	1.26	0–0.5 (6.5%)	0.43
		0.5–1.5 (69%)			0.5–1.5 (67%)	
		1.5–2.5 (25%)			1.5–2.5 (20%)	
		2.5–3.5 (6%)			2.5–3.5 (6.5%)	

OHI II	1.03	0–0.5 (0%) 0.5–1.5 (75%) 1.5–2.5 (25%)	0.44	1.02	0–0.5 (6.7%)	0.38
					0.5–1.5 (80%)	
					1.5–2.5 (6.7%)	
					2.5–3.5 (6.7%)	

OHI III	0.70	0–0.5 (37.5%)	0.27	0.83	0–0.5 (13.2%)	0.38
		0.5–1.5 (56%)			0.5–1.5 (73.5%)	
		1.5–2.5 (6.5%)			1.5–2.5 (13.2%)	

I: first visit, day 1 after the surgery (before CA or CC gel application, baseline).

II: second visit, day 8 after the surgery.

III: third visit, day 22 after the surgery (following CC or CA gel application, final assessment).

**Table 2:** Microorganisms found in the first examination of samples taken from mouth floor mucosa of 16 patients using CA gel with 3% EEP-B.

Isolated microorganisms	Study group CA (first visit, day 1 after the surgery, before CA gel application, baseline)																
	1	2	3	4	5	6	7	8	9	10	11	12	13	14	15	16	All
Gram-positive cocci																	
*Streptococcus mitis*	x		x	x			x		x					x			6
*Streptococcus sanguinis*	x							x				x	x		x	x	6
*Streptococcus salivarius*		x	x		x	x					x						5
*Streptococcus pneumoniae*		x															1
*Staphylococcus epidermidis MSCNS*									x								1
*Ruminococcus productus*															x		1
*Sarcina *sp.							x		x								2

Gram-negative cocci																	
*Neisseria *spp.		x	x	x	x			x				x		x			7

Gram-positive rods																	
*Bifidobacterium adolescentis*											x		x				2
*Bifidobacterium breve*								x									1
*Bifidobacterium dentium*												x					1
*Bifidobacterium infantis*											x						1
*Propionibacterium propionicus*			x														1
*Actinomyces israelii*											x						1
*Actinomyces naeslundii*			x											x			2
*Actinomyces odontolyticus*													x				1
*Actinomyces viscosus*														x			1
*Clostridium perfringens*	x																1
*Corynebacterium *sp.										x							1

Gram-negative rods																	
*Bacteroides distasonis*														x			1
*Campylobacter gracilis*												x					1
*Capnocytophaga ochracea*														x			1
*Escherichia coli*					x		x										2
*Mitsuokella multiacidus*															x		1
*Prevotella bivia*				x													1
*Prevotella melaninogenica*												x	x				2

Fungi																	
*Candida albicans*										x	x						2
*Candida krusei*																x	1

The total number of microbial isolates	3	3	5	3	3	1	3	3	3	2	5	5	4	6	3	2	**54**

x = the presence of a specific microorganism in the examined material.

**Table 3 tab3:** Microorganisms found in the final examination of samples taken from mouth floor mucosa of 16 patients using CA gel with 3% EEP-B.

Isolated microorganisms	Study group CA (third visit, day 22 after the surgery, following CA gel application, final assessment)																
	1	2	3	4	5	6	7	8	9	10	11	12	13	14	15	16	All
Gram-positive cocci																	
*Streptococcus mitis*		x	x		x				x	x					x		6
*Streptococcus oralis*											x						1
*Streptococcus sanguinis*				x			x					x					3
*Streptococcus salivarius*	x					x							x			x	4
*Streptococcus vestibularis*								x									1
*Streptococcus pneumoniae*			x											x			2
*Staphylococcus aureus MSSA*										x							1
*Staphylococcus epidermidis MSCNS*																x	1
*Sarcina *sp.						x	x									x	3

Gram-negative cocci																	
*Neisseria *spp.	x		x	x	x	x	x	x			x	x	x	x	x	x	13
*Veillonella parvula*				x	x		x										3

Gram-positive rods																	
*Bifidobacterium adolescentis*			x														1
*Bifidobacterium dentium*			x														1
*Bifidobacterium longum*		x															1
*Lactobacillus acidophilus*	x																1
*Propionibacterium propionicus*		x															1
*Clostridium ramosum*													x				1

Gram-negative rods																	
*Escherichia coli*					x												1
*Fusobacterium nucleatum*									x								1

Fungi																	
*Candida albicans*										x					x		2

The total number of microbial isolates	3	3	5	3	4	3	4	2	2	3	2	2	3	2	3	4	**48**

x = the presence of a specific microorganism in the examined material.

**Table 4 tab4:** Microorganisms found in the first examination of samples taken from mouth floor mucosa of 15 patients using CC gel without EEP-B.

Isolated microorganisms	Study group CC (first visit, day 1 after the surgery, before CC gel application, baseline)															
	1	2	3	4	5	6	7	8	9	10	11	12	13	14	15	All
Gram-positive cocci																
*Streptococcus mitis*	x		x	x	x		x	x		x		x	x			9
*Streptococcus sanguinis*		x														1
*Streptococcus salivarius*											x					1
*Peptococcus niger*										x						1
*Ruminococcus productus*															x	1
*Sarcina *sp.												x				1

Gram-negative cocci																
*Neisseria *spp.	x	x	x	x			x			x	x	x	x			9
*Veillonella parvula*											x					1

Gram-positive rods																
*Bifidobacterium breve*													x			1
*Bifidobacterium dentium*		x														1
*Bifidobacterium infantis*											x					1
*Propionibacterium acnes*						x		x								2
*Actinomyces israelii*										x				x		2

Gram-negative rods																
*Capnocytophaga ochracea*											x					1
*Escherichia coli*				x					x		x					3
*Mitsuokella multiacidus*													x		x	2
*Prevotella melaninogenica*										x						1

Fungi																
*Candida albicans*				x		x							x			3

The total number of microbial isolates	2	3	2	4	1	2	2	2	1	5	6	3	5	1	2	**41**

x = the presence of a specific microorganism in the examined material.

**Table 5 tab5:** Microorganisms found in the final examination of samples taken from mouth floor mucosa of 15 patients using CC gel without EEP-B.

Isolated microorganisms	Study group CC (third visit, day 22 after the surgery, following CC gel application, final assessment)															
	1	2	3	4	5	6	7	8	9	10	11	12	13	14	15	All
Gram-positive cocci																
*Streptococcus mitis*	x					x	x				x	x		x	x	7
*Streptococcus oralis*			x	x												2
*Streptococcus sanguinis*	x				x			x		x						4
*Streptococcus salivarius*		x		x					x				x			4
*Streptococcus vestibularis*			x													1
*Staphylococcus aureus MSSA*		x														1
*Staphylococcus epidermidis MSCNS*											x					1
*Peptostreptococcus prevotii*					x											1
*Ruminococcus productus*		x														1
*Sarcina *sp.			x					x								2

Gram-negative cocci																
*Neisseria *spp.	x	x	x	x	x	x	x	x	x	x	x		x	x	x	14
*Veillonella parvula*			x		x										x	3

Gram-positive rods																
*Bifidobacterium adolescentis*	x															1
*Lactobacillus acidophilus*							x									1
*Lactobacillus fermentum*										x						1
*Propionibacterium acnes*														x		1
*Actinomyces israelii*	x	x														2
*Actinomyces naeslundii*														x		1
*Actinomyces odontolyticus*								x								1

Gram-negative rods																
*Bacteroides ureolyticus*								x								1
*Campylobacter gracilis*															x	1
*Fusobacterium mortiferum*					x											1
*Fusobacterium nucleatum*				x												1
*Mitsuokella multiacidus*		x														1

Fungi																
*Candida albicans*						x					x	x				3

The total number of microbial isolates	5	6	5	4	5	3	3	5	2	3	4	2	2	4	4	**57**

x = the presence of a specific microorganism in the examined material.

**Table 6:** The number of microorganisms species and strains isolated from mouth floor mucosa of patients, who used CA gel (with 3% EEP-B) or CC gel (without EEP-B).

Specimen	First examination		Final examination	
	Species	Strains	Species	Strains
CA gel	28	54	20	48
CC gel	18	41	25	57
